# Albumin Synthesis in States of Inflammation

**DOI:** 10.1186/2197-425X-3-S1-A42

**Published:** 2015-10-01

**Authors:** A Komáromi, F Hammarkvist, O Rooyackers, J Wernerman, Å Norberg

**Affiliations:** Karolinska University Hospital Huddinge, Anaesthesia and Intensive Care, Stockholm, Sweden; Karolinska University Hospital Huddinge, Surgery, Stockholm, Sweden; Karolinska Institutet, CLINTEC, Anaesthesia, Stockholm, Sweden

## Introduction

Plasma albumin concentration is a negative acute phase reactant, decreasing in critical illness, with acute inflammation, and in connection with major surgical procedures where inflammation is a likely contributor. How this depletion of plasma albumin relates to albumin synthesis rate is poorly investigated.

## Objectives

To study albumin synthesis rate in relation to acute inflammation in humans.

## Methods

Healthy volunteers in the postabsorptive state (n = 10), patients with acute inflammatory abdominal disease and plasma C-reactive protein over 100 mg/L (n = 10), and patients scheduled for elective pancreatic resection at the beginning of surgical procedure (n = 10) were studied. The albumin synthesis rate was measured by the flooding dose technique comprising the incorporation of deuterium-labeled phenylalanine in de novo synthesized albumin determined by a gas chromatography mass spectrometer.[1] Total albumin pool was determined by measuring plasma volume using ^125^I-albumin and plasma albumin concentrations. The 3 groups were compared by means of one-way analysis of variance.

## Results

Absolute synthesis rate of albumin was 118 ± 16, 180 ± 57 and 96 ± 25 mg/kg/day, in the control, the acute inflammatory, and pancreatic surgical groups, respectively (p < 0.001). Plasma albumin concentrations at the start of measurements was 39.8 ± 3.4, 25.7 ± 5.5 and 27.9 ± 5.2 g/L (p < 0.001). This corresponded to fractional synthesis rate of albumin of 7.4 ± 1.1, 17.1 ± 3.8 and 8.5 ± 1.4 % per day of the intravascular albumin pool, respectively (p < 0.001).

## Conclusions

A more than 50% elevation of de novo albumin absolute synthesis rate was seen in patients with acute abdominal inflammation compared to volunteers and patients scheduled for pancreatic surgery. The synthesis rate for albumin did not differ between the volunteers and patients scheduled for surgery, despite the lower plasma albumin concentrations in the latter group. This might relate to their underlying pathology associated with a chronic inflammation.

## Grant Acknowledgment

Swedish Medical Research Council, and the Country Council of Stockholm.
